# Anesthetic management of cesarean delivery for a parturient with Wilson's disease: A case report

**DOI:** 10.1097/MD.0000000000010454

**Published:** 2018-05-18

**Authors:** Yantong Wan, Xiaoqin Jiang, Xuemei Lin

**Affiliations:** Department of Anesthesiology, West China Second University Hospital, Key Laboratory of Birth Defects and Related Diseases of Women and Children, Sichuan University, Ministry of Education, Chengdu, Sichuan, People's Republic of China.

**Keywords:** anesthetic management, cesarean delivery, Wilson's disease

## Abstract

**Rationale:**

Wilson's disease (WD), or hepatolenticular degeneration, is an autosomal recessive disorder with a prevalence of 1:50,000 to 1:100,000 live births.

**Patient concerns:**

A 26-year-old primipara with WD was admitted to our hospital, due to awaiting delivery. Her main symptoms were slightly higher total bile acid (TBA) and bilateral depressed edema of lower limbs.

**Diagnosis:**

She was at 38 weeks and 4 days of gestation with a 15-year history of WD, controlled with penicillamine in the past and replaced by zinc preparations from three months before pregnancy.

**Outcomes:**

General anesthesia was successfully administered for a female with WD undergoing cesarean delivery.

**Lesson:**

General anesthesia can be administered in an asymptomatic primigravida with WD. Appropriate anesthetics choice can effectively minimize the rates of complications and sequelae.

## 1 Introduction

Wilson's disease [(WD), or hepatolenticular degeneration, is an autosomal recessive disorder with a prevalence of 1:50,000 to 1:100,000 live births.^[1]^ The metabolism disturbance of copper for lack of ceruloplasmin leads to neuropsychiatric and hepatic symptoms such as tremor, seizures, chronic hepatitis, and even acute liver failure.^[2]^ The excessive copper accumulation in the uterus of women results in poor pregnancy rate and recurrent miscarriage of women with WD.^[1]^ Zinc sulphate as an effective therapy could be helpful for successful pregnancy outcomes. The maternal serum copper level may decline modestly during pregnancy probably due to fetal intake of copper from the maternal serum.^[1]^ Liver injuries and cirrhosis make challenges to the choices of anesthetic techniques and anesthetic drugs. Here, we report a case of a successful childbirth in a female with WD.

## 2 Case report

A 26-year-old primipara was admitted to our hospital, due to awaiting delivery. She was at 38 weeks and 4 days of gestation with a 15-year history of WD, controlled with penicillamine in the past and replaced by zinc preparations from 3 months before pregnancy. Her laboratory results of liver function on a regular basis were normal owing to regular medication. Penicillamine caused teratogenic effects on patients but zinc therapy has no teratogenic effects and is optimal treatment for the pregnant with WD.^[3]^ The laboratory examination of low serum ceruloplasmin and ascites led to the diagnosis of WD and cirrhosis 15 years ago. On admission both the vital signs of the parturient and the fetal movement were normal. The obstetrics ultrasound suggested a loop of cord had been round the neck of the fetus for one week. Her main symptoms were slightly higher total bile acid (TBA) and bilateral depressed edema of lower limbs. Laboratory investigations were within the normal ranges excluding total bilirubin (TB): 27.3 umol/L, platelet: 56×10^9/L. Ultrasound abdomen revealed asymmetry of liver echotexture and splenomegaly.

At the request of the patient and her family, the cesarean delivery was operated on the primigravida at 39 weeks of gestation. Owing to the decrease of the platelet in previous investigations, general anesthesia was administered for the parturient. General anesthesia was induced with intravenous propofol 100 mg, remifentanil 70 ug, and succinylcholine 100 mg in rapid sequence, followed by trachea intubation with glidescope. The anesthesia was maintained with 2% inhaled sevoflurane at fresh gas flow of 2 L/min. ETCO_2_ was maintained between 30 to 38 mmHg. The fetus was delivered 2 minutes after incision, with Apgar scores of 8 at 1 and 9 at 5 minutes. Midazolam 2 mg, sufentanil 15 ug, and along with atracurium 10 mg was given intravenously. The rapid blood gas analysis of the neonate revealed SPO2: 65%. The appropriate measures including keeping warm, airway clearance, chest compression, and balloon positive pressure ventilation were performed at once. The patient was extubated without any problems and taken to the ward. The patient received continuous pump infusion of tramadol 0.4 mg/kg/h intravenously to control pain after delivery. The routine blood test on the first postoperative day was within normal limits except HGB: 107 g/L, platelet: 50^×^10^9/L. The postoperative course was uneventful and the patient was discharged on the third day.

## 3 Discussion

There are a few case reports of successful pregnancy outcomes and childbirths undergoing spontaneous labor.^[1]^ General anesthesia for WD patients undergoing cesarean delivery was rarely reported. Anesthetics choices in general anesthesia for gravidas with WD make challenges to anesthetists. In the anesthesia management of patients with WD, the impairment of liver and kidney influences the metabolism of anesthetics.^[4]^ The major concern was to ensure the safety of the mother and the infant and to reduce the use of anesthetics metabolized by liver. Moreover, WD patients may be more sensitive to neuromuscular relaxants for the disease itself or for the use of penicillamine.^[5]^ Atracurium may be the better choice for WD since it is metabolized by Hofmann elimination, not dependent on hepatic or renal function. However, the onset time and effect elimination time of atracurium for anesthesia induction were longer than those of succinylcholine. So succinylcholine is unavoidably used for rapid sequence induction. Both succinylcholine rarely crosses placental barrier and has less impacts on the fetus with a rapid onset of action and shorter half-life. There were no drug residues on patient and adverse effects on the fetus postoperatively according to our observation and the parturient awaked up quickly. The combination of propofol and remifentanil cut down the hepatic impact for extra-hepatic elimination ways.^[4]^ The use of remifentanil was advised in high-risk patients for stable hemodynamic and prevalence of intraoperative awareness.^[6]^ Propofol reduces the cardiovascular response to laryngoscopy and tracheal intubation.^[7]^

In summary, for parturient with WD, epidural and spinal anesthesia can be achieved for those who have no contraindications to regional anesthesia. Otherwise, general anesthesia is the better choice. Appropriate anesthetics choice can effectively minimize the rates of complications and sequelae.

## Author contributions

**Data curation:** Xiaoqin Jiang.

**Writing – original draft:** Yantong Wan.

**Writing – review & editing:** Xuemei Lin.
